# Supplementary material for: Thrombin Preconditioning Enhances Therapeutic Efficacy of Human Wharton’s Jelly–Derived Mesenchymal Stem Cells in Severe Neonatal Hypoxic Ischemic Encephalopathy
Source: Int J Mol Sci. 2019 May 20;20(10):2477. doi: 10.3390/ijms20102477 (PMC6566845; doi:10.3390/ijms20102477)
Supplement: Supplementary file 1 [file ijms-20-02477-s001.pdf]

Supplementary Materials

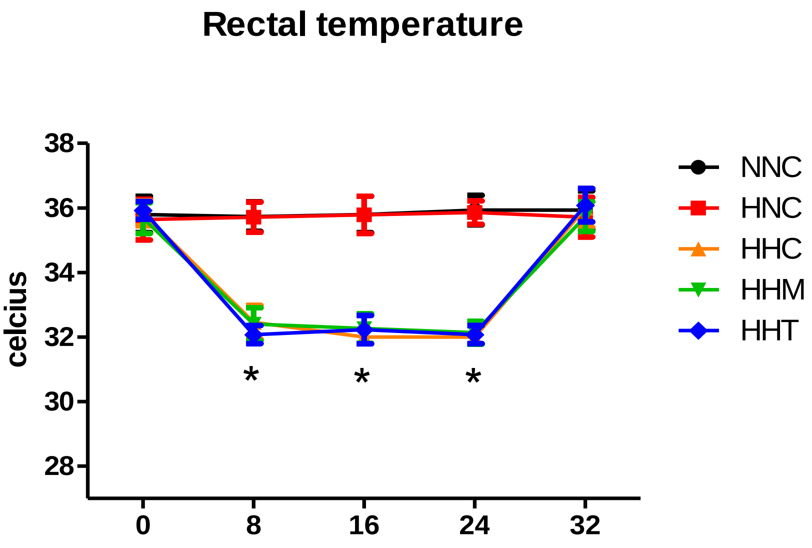

Supplementary Figure S1. Rectal temperature in experimental groups. Temperatures in each group remained stable during the intervention and were significantly different between normothermia and hypothermia groups at each measurement. n = 19, 45, 30, 44 and 40, NNC, HNC, HHC, HHM and HHT, respectively. Data are mean ± SD. NNC, normal+normothermia control; HNC, HIE + normothermia control; HHC, HIE + hypothermia control; HHM, HIE + hypothermia + naïve MSCs; HHT, HIE + hypothermia + thrombin-primed MSCs. * $P < 0.05$ vs. normothermia groups (NNC and HNC).
